# Management of a Hospital-Wide COVID-19 Outbreak Affecting Patients and Healthcare Workers

**DOI:** 10.1007/s42399-020-00597-2

**Published:** 2020-10-26

**Authors:** Steffen Höring, René Fussen, Johannes Neusser, Michael Kleines, Thea Laurentius, Leo Cornelius Bollheimer, Doris Keller, Sebastian Lemmen

**Affiliations:** 1 Division of Infection Control and Infectious Diseases, Medical Faculty, RWTH Aachen University Hospital, Aachen, Germany; 2 Laboratory Diagnostic Center, RWTH Aachen University Hospital, Aachen, Germany; 3 Department of Geriatric Medicine, RWTH Aachen University Hospital, Aachen, Germany; 4 University Medical Center for Occupational Medicine, RWTH University, Aachen, Germany

**Keywords:** COVID-19, Infection control, SARS-CoV-2, Nosocomial outbreak

## Abstract

To the best of our knowledge, here, we describe the first hospital-wide outbreak of SARS-CoV-2 that occurred in Germany in April 2020. We aim to share our experience in order to facilitate the management of nosocomial COVID-19 outbreaks in healthcare facilities. All patients and hospital workers were screened for SARS-CoV-2 repeatedly. An infection control team on the side was installed. Strict spatial separation of patients and intensified hygiene training of healthcare workers (HCW) were initiated. By the time of reporting, 26 patients and 21 hospital workers were infected with a cluster of cases in the geriatric department. Fourteen patients developed COVID-19 consistent symptoms and five patients with severe pre-existing medical conditions died. The outbreak was successfully contained after intensified infection control measures were implemented and no further cases among patients were detected over a period of 14 days. Strict application of standard infection control measures proved to be successful in the management of nosocomial SARS-CoV-2 outbreaks.

**Electronic supplementary material:**

The online version of this article (10.1007/s42399-020-00597-2) contains supplementary material, which is available to authorized users.

## Introduction

Since the beginning of the novel coronavirus disease pandemic (COVID-19), inadvertent exposure of hospitalized patients and HCW to severe respiratory syndrome coronavirus 2 (SARS-CoV-2) has been a major concern [1]. Here, we report a large nosocomial outbreak of SARS-CoV-2 that occurred at a satellite hospital of the University Hospital Aachen, Germany, with 26 patients and 21 healthcare workers infected. The hospital, a formerly church-run facility, was integrated to the University Hospital in January 2020, hosting a geriatric department, a dermatological ward, and a mixed ward for multiple surgical disciplines with 170 beds in total. Located in Aachen, the hospital is situated in close proximity to the district of Heinsberg, the region where community transmission of SARS-CoV-2 was first observed in Germany and where the cumulative incidence of SARS-CoV-2 is still among the highest in Germany [2].

Our report provides a narrative description of a nosocomial COVID-19 outbreak. Furthermore, we present the infection control measures implemented to contain the outbreak and describe potential sources of the outbreak.

## Outbreak Description

Against the background of the ongoing COVID-19 pandemic, the hospital’s policy and clinical processes were already adapted to prevent nosocomial transmission of SARS-CoV-2. All HCW were obliged to wear a surgical face mask throughout their working hours and visitors were no longer permitted. Furthermore, the University Hospital’s peripheral and intensive care capacities were steadily increased, for instance by postponing elective surgeries, in expectation of a rise in COVID-19 case numbers.

In this context, the first SARS-CoV-2-infected patient was revealed in the geriatric department on April 5. Although the patient, a 82-year-old man, showed no signs of infection, polymerase chain reaction (PCR) testing was performed since public health regulations demanded SARS-CoV-2 testing prior to his admission to a long-term care facility.

As the hospital’s pandemic policies required a reduced operation mode, only 50 patients were present at the hospital at the time the first patient was detected. Hence, the potential index patient and all contact patients could be transferred immediately to single rooms. In addition, all patients of the geriatric department were screened for SARS-CoV-2 by nasopharyngeal swabs and PCR analysis (Realstar® SARS-CoV-2 RT-PCR Kit, Altona Diagnostics, Germany) on April 7. The screening revealed ten more oligosymptomatic SARS-CoV-2-infected patients on two spatially separated geriatric wards.

Facing this high detection rate, cohorting of patients on a separated isolation ward was initiated by the infection control department on the same day.

In parallel, on April 6, a 47-year-old nurse working in the geriatric department presented at the University Hospital’s COVID-19 screening center. During the absence from work, she had developed mild symptoms with fever, dry cough, and myalgia outside and was subsequently tested positive for SARS-CoV-2. The nurse belonged to a religious community of seven nuns, all of them working as nurses in the hospital. The remaining six nurses presented the next day at the screening center reporting headache and faintness. In retrospect, the mild symptoms were considered COVID-19-consistent. PCR testing revealed five of them as SARS-CoV-2-positive.

Considering the numerous COVID-19 cases among patients and HCW, a hospital-wide screening was initiated on April 8 for all remaining SARS-CoV-2-negative patients and entire hospital staff. This hospital-wide screening revealed five more cases among patients as well as six nurses, one physiotherapist, and one resident of the dermatology department. While the cases were distributed in departments all over the hospital, there was a cluster among patients and HCW in the geriatric department. Follow-up screening of all SARS-CoV-2-negative patients and hospital staff was conducted repeatedly.

The first follow-up screening between April 11 and April 16 revealed ten more infected patients, four more cases among nursing staff, and two infected occupational therapists. The second follow-up screening between April 20 and 21 yielded one more infected hospital employee, a pastoral worker, but no new cases among patients. The last screening sessions, conducted on April 23/24 and April 27/28, revealed no further cases among patients or hospital workers.

In total, 150 PCR analyses were conducted on 50 patients and 701 PCR analyses were conducted on 270 hospital workers during the observational period. By the time of reporting (May 5), 26 out of 50 patients and 21 out of 270 tested HCW were infected, resulting in an attack rate of 52% and 7.8%, respectively. The median age of patients affected was 85 years and all patients had at least one or even several underlying diseases. Twenty-two of the 26 affected patients were patients of the geriatric department; the remaining four were dermatological and orthopedic patients.

**Fig. 1:**
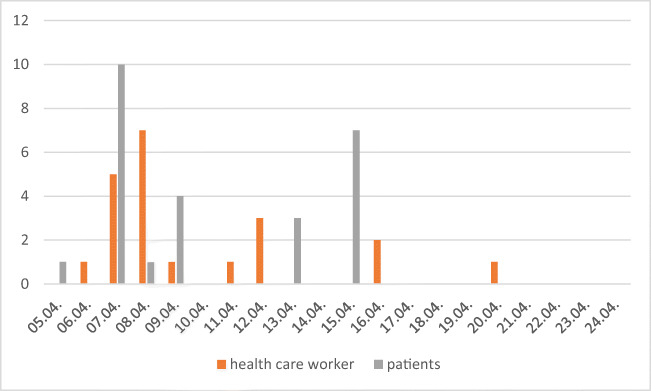
SARS-CoV-2-positive PCR results in the course of the outbreak

Figure 1 demonstrates the occurrence of cases among patients and HCW during the outbreak period. While twelve patients were asymptomatic, nine patients developed COVID-19 with mild to moderate symptoms. However, five elderly patients (mean age 82.2 years; range: 64–94 years) with severe pre-existing conditions (mean Charlson comorbidity index 5.6; range: 4–7) succumbed to the infection; according to their wishes, no life-supporting measures were undertaken. Thus, a case fatality rate of 19.2% (5 out of 26 patients) among infected patients was observed. Among healthcare workers, one nurse was hospitalized due to worsening of her general condition and respiratory distress. By the time of reporting, she had been discharged and has recovered from the infection.

## Infection Control Measures

On April 7, an isolation ward with 40-bed capacity was established for confirmed SARS-CoV-2 cases. On this ward, intensified clinical monitoring was conducted by measuring vital signs and oxygen saturation at least six times a day. Infection control personnel (ICP) and an infectious diseases doctor were present during ward rounds to observe working processes and to support clinical decision-making on a daily basis. The ward facilities were partially restructured with a sluice area and a changing room at the entrance. In the time before the outbreak, it was common practice for nursing staff to work on different wards and to switch their deployments from day to day. With the implementation of the isolation ward, medical staff exclusively worked on one ward without intrahospital fluctuation.

A permanent on-site outbreak team was installed on April 8. The team met daily and consisted of members of the infectious disease and infection control department, the geriatric department, head nurses, and the hospital’s managing director.

Throughout the outbreak, all hospital workers wore a surgical face mask during their working hours. When caring for SARS-CoV-2-positive patients, gloves, goggles, and a protective gown were worn additionally and an FFP-2 face mask was used whenever aerosol-producing procedures were expected. In order to ensure safe handling of the personal protection equipment, i.e., preventing self-inoculation during ungloving, gowning, and masking, the medical staff was trained by ICP repeatedly.

Since April 8, the geriatric clinic was closed for new admissions, and from April 17 on, this regulation was applied for the entire hospital. Spatial distancing was additionally ensured by keeping all SARS-CoV-2-negative patients in single rooms outside the isolation ward.

As outlined above, SARS-CoV-2 screening of patients and hospital staff was performed twice weekly by nasopharyngeal swabs and PCR analysis. Examination of hospital workers, including HCW as well as administrational or technical personnel, was conducted as a voluntary mass screening during working hours. SARS-CoV-2-positive staff were released from work and put under domestic quarantine until symptoms entirely for at least 48 h and PCR testing proved negative twice in a row.

## Investigation of the Outbreak Source

Our analysis of the intrahospital SARS-CoV-2 transmission dynamics is based on time of diagnosis, time of admission, time of onset of symptoms, viral load at initial PCR testing, and reported contacts of persons infected (see supplementary Figs. 1 and 2). PCR-derived threshold cycle (Ct) values served as a surrogate for viral load with low Ct values below 20 indicating a high viral load and Ct values above 30 representing a low viral load.

On the one hand, there was the first case among patients, detected on April 5. The high viral load (Ct: 16) at the time of diagnosis and the fact that symptoms worsened after diagnosis point to a recently acquired COVID-19 infection. At the time of diagnosis, the patient was already hospitalized for several weeks. Due to colonization with multi-resistant bacteria, single-room isolation precautions were already performed; thus, no patient-to-patient contact occurred during the hospital stay. Following the hospitals’ pandemic regulations, no visitors were allowed in the preceding weeks. Therefore, we conclude a nosocomial infection transmitted via an infected HCW.

Considering the date of admission and onset of symptoms, a further 21 cases among patients are categorized as nosocomial infections. However, in one case, SARS-CoV-2 virus was detected only 2 h after hospital admission pointing to a community-acquired infection. In three other cases, the date of admission was within the assumed incubation period; thereby, no definite mode of acquisition can be stated for these patients.

On the other hand, we analyzed the first cases among hospital staff, starting with the potential index nurse tested positive for SARS-CoV-2 on the 6th of April. Low Ct values and worsening of symptoms in the days after diagnosis suggest a recently acquired COVID-19 infection with a high potential for viral spreading. Five of her household members, all of them nuns living together in a religious community, were infected showing lower viral loads but a simultaneous onset of symptoms. Contacts to patients and other hospital workers could not be traced back reliably since all affected nurses shifted teams and wards frequently within the hospital on a needs basis. No definite index case or source of infection can be determined for this cluster.

## Discussion

This report presents our first experience in managing a nosocomial COVID-19 outbreak. In total, 26 patients and 21 HCW were infected with SARS-CoV-2. Since mainly elderly patients with severe pre-existing medical conditions were affected, a high case fatality rate of 19% was observed during the outbreak. Nevertheless, intensified infection control measures eventually led to successful containment.

The outbreak occurred during the onset of the COVID-19 pandemic in Germany. By the time the outbreak emerged, the hospital policy already comprised preemptive infection control measures in order to prevent intrahospital spread of SARS-CoV-2. Nevertheless, the outbreak could only be contained after all potential routes of intrahospital virus transmission were addressed by additional infection control measures (Table 1).

- First, the patient-to-patient transmission of SARS-CoV-2 had to be prevented. The reduction of contacts between geriatric patients was partially challenging since several patients suffered from cognitive impairment and did not follow social distancing recommendations or single-room isolation. Thus, cohorting of infected patients on an isolation ward proved to be an ideal solution in this scenario. Patients could move freely within the limits of the isolation ward and social contacts between patients were permitted without putting non-infected patients at risk.

**Table 1:** Outbreak control measures

• Establishment of a multidisciplinary outbreak team • Establishment of an isolation ward • Single room placement of uninfected patients • Rejection of new admissions • Intensified clinical monitoring of COVID-19 patients • Serial PCR screening of patients and hospital employees • Employment leave for SARS-CoV-2-infected staff • Training of healthcare workers • Outbreak investigation	

The second route of transmission addressed by our measures was infected HCW, who potentially spread SARS-CoV-2 to patients as well as to their co-workers. On the one hand, geriatric care requires close physical contacts thereby facilitating viral spreading from HCW to patients. Consistently, first reports on outbreaks in nursing homes and geriatric wards show high attack rates and transmission dynamics comparable to our outbreak scenario [3–5]. We assume that by identifying asymptomatic SARS-CoV-2-infected patients and officially declaring the circumstances a nosocomial outbreak, not only personal protective equipment (PPE) was used more consequently but also HCWs’ practice of care, for example avoiding close face-to-face contact with infected patients, might have altered temporarily. On the other hand, the viral spread might also occur between HCW. In our case, for instance, HCW occasionally reported not to have worn facemasks during breaks although spatial distance could not be kept in these situations. Both routes of transmission, HCW to patients and HCW to HCW, were successfully addressed in infection control training sessions in which HCW were instructed in the correct handling of personal protective equipment and in social distancing measures. Several reports point out the importance of verbal training sessions demonstrating that HCW education does not only improve the handling of PPE but also reduces anxiety and increases the sense of preparedness [6, 7].

Last, our measures aimed to prevent the introduction of new COVID-19 cases into the hospital. Thus, the hospital was closed for new admissions during the ongoing outbreak. In the post-outbreak period, we have continued to screen all patients on their day of admission and all geriatric inpatients once weekly for SARS-CoV-2 in order to detect new cases timely. Serial screening proved necessary since detection of a newly acquired COVID-19 infection cannot reliably be achieved by a single PCR test. Viral RNA shedding starts approximately a day before the onset of symptoms and peaks in the first week of the disease with no detectable RNA in the first days post infection [8–10]. This explains why, in our case, seven HCW were initially tested negative, while follow-up examinations revealed a COVID-19 infection.

Eventually, the infection control measures undertaken in response to the outbreak (Table 1) turned out to be effective since no further case among patients was detected after April 15 and only one last case among healthcare workers occurred on April 20. This report emphasizes the necessity of an infection control team on-site in an outbreak situation. Unlike many German hospitals, where infection control specialists are not present permanently, a well-established infection control infrastructure with sufficient manpower was available in our case.

Concerning the source of this outbreak, patient-to-patient contacts did not seem to be the main factor. Most affected patients were bedridden and had no contact with other patients. Nevertheless, three patients suffering from dementia showed a tendency to wander around on the wards and entered other patients’ rooms without permission. This behavior might have led to SARS-CoV-2 transmissions in singular cases in the weeks preceding the outbreak.

A greater contribution to outbreak dynamics might have been made by infected, asymptomatic or paucisymptomatic HCWs. Although we have not revealed any erroneous infection control behavior on the side of HCWs, we assume they played a crucial role in introducing and spreading SARS-CoV-2 in the hospital. The cluster observed among HCWs in the religious community serves as a good example of this assumption. The order of nuns lived under circumstances that clearly fostered viral transmission. They stayed together in a dormitory on the hospital’s premises, shared a household, and attended service together. All of them simultaneously developed symptoms pointing to a commonly acquired infection, most likely outside their working hours. However, all of them were still employed in patient care during their assumed incubation period and therefore could have introduced and spread the virus on the wards as asymptomatic carriers. We therefore hypothesize that SARS-CoV-2 might have initially spread and incubated among HCWs and was subsequently transmitted to patients and further co-workers.

Nevertheless, we must discuss alternative ways of SARS-CoV-2 introduction to the hospital. In one patient for example, a community-acquired infection was clearly given. Therefore, we assume that the outbreak was based on multiple routes of introduction and transmission. Eventually, a definite single outbreak source could not be determined.

Further molecular investigation, e.g., next-generation sequencing (NGS), might have been useful to clarify infection chains retrospectively. Nevertheless, we state that no additional benefits for the actual outbreak management would have derived from further molecular diagnostics since the outbreak dynamics obviously suggested a nosocomial spread of SARS-CoV-2 in our case. We therefore claim that our report emphasizes the sufficiency of standard diagnostic methods under the exceptional circumstances of a nosocomial SARS-CoV-2 outbreak.

There are exceptional infrastructural aspects that clearly facilitated the outbreak management in our case. Although the hospital affected was small with a bed capacity of 170 beds only, as a satellite hospital, it could fall back on the infrastructure and the financial power of a large tertiary care university hospital. This ideal setting not only allowed the closure of the entire facility to new admissions but also provided fast diagnostic processes with same day PCR results and an infection control team on site. We are aware of the fact that these settings are not a common standard and therefore, the management of SARS-CoV-2-outbreaks can be more difficult for other hospitals and healthcare facilities.

Finally, we plead for a frank and detailed communication of nosocomial SARS-CoV-2 outbreaks during the ongoing COVID-19 pandemic. Despite concerns of negative publicity, reporting of nosocomial outbreaks is essential to allow all parties in the healthcare sector to benefit from each other’s experience.

## Conclusion

Our report demonstrates a successful containment strategy for nosocomial COVID-19 outbreaks. Multiple routes of transmission and delayed PCR-based diagnosis of early-stage infections presented pitfalls and hampered the definite investigation of the outbreak. Nevertheless, routine diagnostics and standard infection control measures, e.g., contact precautions and screening of patients and HCW, proved to be efficient when applied to this novel pathogen and allowed successful outbreak management.

## Electronic Supplementary Material

ESM 1 (DOCX 24 kb)

ESM 2 (DOCX 26.5 kb)
