# Supplementary material for: Management of a Hospital-Wide COVID-19 Outbreak Affecting Patients and Healthcare Workers
Source: SN Compr Clin Med. 2020 Oct 26;2(12):2540–5. doi: 10.1007/s42399-020-00597-2 (PMC7586206; doi:10.1007/s42399-020-00597-2)
Supplement: Supplementary file 1 [file 42399_2020_597_MOESM1_ESM.docx]

April

Fallnummer	Erstnachweis	ct-Wert	1	2	3	4	5	6	7	8	9	10	11	12	13	14	15	16	17	18	19	20	21	22	23	24	25	26	27	28	29	30
Index case	Geriatrics	16	<<																													>>
Case #2	Geriatrics	20	<<																							>						
Case #3	Geriatrics	19	<<																													>>
Case #4	Geriatrics	23	<<																							>						
Case #5	Geriatrics	12		<																												>>
Case #6	Geriatrics	15	<<																													>>
Case #7	Geriatrics	31		<																						>						
Case #8	Geriatrics	13	<<															T		#												
Case #9	Geriatrics	14	<<																													>>
Case #10	Geriatrics	23	<<											T		>								<								>>
Case #11	Geriatrics	16							<			T																	#			
Case #12	Geriatrics	30	<<														#															
Case #13	Dermatology	33		<																						>						
Case #14	Orthopaedic surgery	36	<<									T																>				
Case #15	Orthopaedic surgery	34							<			>																				
Case #16	Dermatologie	31							<			>							<													>>
Case #17	Geriatrics	18		<																				T						#		
Case #18	Geriatrics	32							<													#										
Case #19	Geriatrics	30							<																						>	
Case #20	Geriatrics	31	<<																													>>
Case #21	Geriatrics	31								<								T													>	
Case #22	Geriatrics	32			<																											>>
Case #23	Geriatrics	32	<<																													>>
Case #24	Geriatrics	32					<																									>>
Case #25	Geriatrics	32							<																							>>
Case #26	Geriatrics	30	<<																													>>

negative PCR result

positive PCR result

symptomatic

hospital stay

<<>> admission/discharge outside observation period

T transfer

deceased

< > admission/discharge
