# Supplementary material for: Management of a Hospital-Wide COVID-19 Outbreak Affecting Patients and Healthcare Workers
Source: SN Compr Clin Med. 2020 Oct 26;2(12):2540–5. doi: 10.1007/s42399-020-00597-2 (PMC7586206; doi:10.1007/s42399-020-00597-2)

	Department	Profession	ct-value
Index case	Geriatrics	Nurse	17
Case #2	Geriatrics	Nurse	24
Case #3	Geriatrics	Nurse	24
Case #4	Geriatrics	Nurse	29
Case #5	Geriatrics	Nurse	32
Case #6	Geriatrics	Nurse	23
Case #7	Geriatrics	Nurse	27
Case #8	Geriatrics	Nurse	33
Case #9	Geriatrics	Nurse	22
Case #10	Geriatrics	Nurse	32
Case #11	Geriatrics	Nurse	21
Case #12	Physiotherapy	Physiotherapsit	19
Case #13	Geriatrics	Nurse	21
Case #14	Dermatology	Resident	33
Case #15	Occupational therapy	Occupational therapist	n.a.
Case #16	Geriatrics	Nurse	35
Case #17	Geriatrics	Nurse	33
Case #18	Geriatrics	Nurse	32
Case #19	Occupational therapy	Occupational therapist	30
Case #20	Geriatrics	Nurse	28
Case #21	Pastoral care	Pastoral worker	23

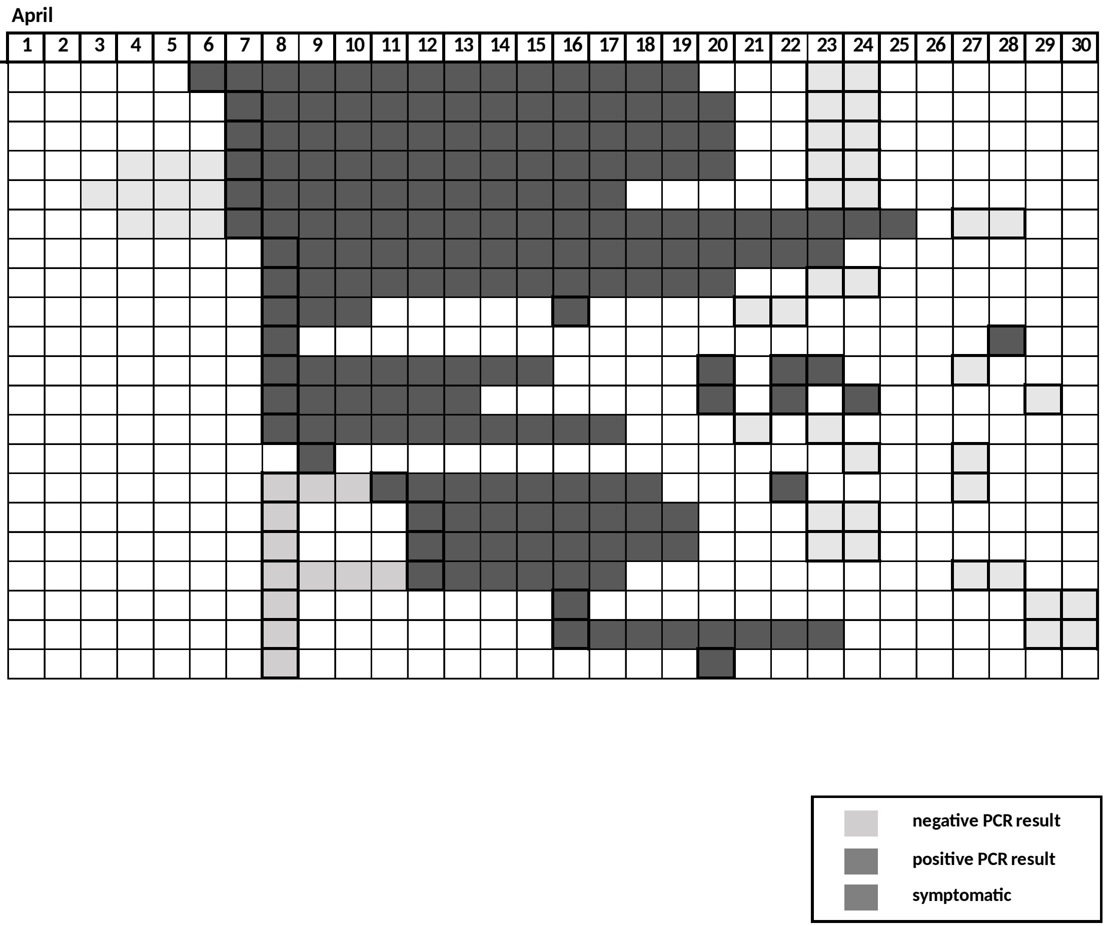

Supplement: Supplementary file 2 [file 42399_2020_597_MOESM2_ESM.docx]
